# Third-Generation Vaccines: Features of Nucleic Acid Vaccines and Strategies to Improve Their Efficiency

**DOI:** 10.3390/genes13122287

**Published:** 2022-12-04

**Authors:** Alanne Rayssa da Silva Melo, Larissa Silva de Macêdo, Maria da Conceição Viana Invenção, Ingrid Andrêssa de Moura, Marco Antonio Turiah Machado da Gama, Cristiane Moutinho Lagos de Melo, Anna Jéssica Duarte Silva, Marcus Vinicius de Aragão Batista, Antonio Carlos de Freitas

**Affiliations:** 1 Laboratory of Molecular Studies and Experimental Therapy—LEMTE, Department of Genetics, Federal University of Pernambuco, Recife 50670-901, Brazil; 2 Laboratory of Immunological and Antitumor Analysis, Department of Antibiotics, Bioscience Center, and Keizo Asami Imunophatology Laboratory, Federal University of Pernambuco, Recife 50670-901, Brazil; 3 Laboratory of Molecular Genetics and Biotechnology (GMBio), Department of Biology, Center for Biological and Health Sciences, Federal University of Sergipe, São Cristóvão 49100-000, Brazil

**Keywords:** vaccines, nucleic acids, synthetic genes, adjuvants

## Abstract

Gene immunization comprises mRNA and DNA vaccines, which stand out due to their simple design, maintenance, and high efficacy. Several studies indicate promising results in preclinical and clinical trials regarding immunization against ebola, human immunodeficiency virus (HIV), influenza, and human papillomavirus (HPV). The efficiency of nucleic acid vaccines has been highlighted in the fight against COVID-19 with unprecedented approval of their use in humans. However, their low intrinsic immunogenicity points to the need to use strategies capable of overcoming this characteristic and increasing the efficiency of vaccine campaigns. These strategies include the improvement of the epitopes’ presentation to the system via MHC, the evaluation of immunodominant epitopes with high coverage against emerging viral subtypes, the use of adjuvants that enhance immunogenicity, and the increase in the efficiency of vaccine transfection. In this review, we provide updates regarding some characteristics, construction, and improvement of such vaccines, especially about the production of synthetic multi-epitope genes, widely employed in the current gene-based vaccines.

## 1. Introduction

Throughout history, we have experienced how several disease outbreaks have caused health risks, many of them with pandemic potential, culminating in the deaths of millions of people worldwide. The emergence of new diseases accompanied by population growth and globalization indicates the need to obtain new tools capable of reducing the transmission of infectious agents and the risk of future pandemics [1,2]. In this context, vaccines represent a valuable measure for maintaining global health, offering protection, and contributing to the control and combat of several pathogens that threaten human and veterinary health [3]. More than two centuries after the creation of the first vaccine, the field of vaccinology has promoted the improvement of classic immunization techniques. These approaches include use of attenuated or inactivated pathogens, or even toxoids, and the creation and application of new strategies, such as live vectors and nucleic acids [4].

In 2020, during the COVID-19 pandemic scenario caused by SARS-CoV-2, the urgent nature of protective measures prompted rapid action by the scientific community through pre-existing studies [5]. In particular, platforms based on nucleic acids (DNA and mRNA) stood out in the face of the urgency caused by COVID-19 and presented an unprecedented milestone in the history of vaccines due to their licensing for emergency use in humans. The antigens used in these vaccine strategies have been obtained and designed through reverse and structural vaccinology techniques and bioinformatics tools in which data from in silico validated pathogens, proteins or peptides are used, for example, for the construction of vaccine antigens [6].

As seen in recent epidemic outbreaks, future pandemics are likely to require the continued development of new models and approaches for designing nucleic acid vaccines. Particularly with viral infections, there is a demand for rapid production and updating of vaccine platforms. These improvements are essential not only for diseases not controlled through vaccination, but also in the context of the appearance of mutations that lead to the emergence of variants or the establishment of new serotypes. In addition, it is also essential to invest in the vaccine targets’ presentation, which can be whole genes or constructs based on epitopes predicted in silico, besides the development of adjuvants and immunomodulators [6,7]. Therefore, this research field is continuously expanding, especially regarding third-generation vaccines. In this review, we focus on developing the constructs and improving the efficiency of nucleic acid vaccines, emphasizing the design of synthetic multi-epitope antigens.

## 2. New Technologies: Gene-based Vaccines

Genetic vaccines consist of immunizing or immunotherapeutic approaches that employ DNA or RNA plasmids as antigen precursors. The gene sequence (of one or more genes) encoding the antigen of interest is taken up and translated into protein by host cells. This so-called third-generation vaccine technology, considered innovative among vaccine platforms, was widely used in the COVID-19 immunization program and has great potential to become increasingly common. In addition, these vaccines can trigger cellular and humoral responses and can be improved through in silico tools, which allow the selection of antigenic epitopes, called synthetic antigen vaccines.

The manufacture of vaccines that have RNA or DNA molecules in their composition dispenses with the large-scale cultivation of pathogenic microorganisms in a laboratory with a high level of biosafety, such as BSL3, to the detriment of conventional vaccine strategies, such as those using attenuated or inactivated vaccines. In addition, the absence of the pathogen prevents the virus reactivation. This aspect favors the vaccination of immunocompromised people [8].

Indeed, nucleic acid vaccines also exhibit significant advantages over traditional vaccines regarding their ability to induce CD4+ and CD8+ T cell responses. The inherent immunostimulatory nature of the mRNA molecule and its functionality as an immunoadjuvant is regarded as a strength that can be exploited in vaccine strategies. Transforming these characteristics into a safe and effective clinical product presents the challenge of balancing immune stimulation with the expression of the encoded antigen. Lately, mRNA vaccines have received special attention and exhibit some advantages over DNA vaccines, such as targeting delivery only to the cell cytoplasm, nullifying the risk of genomic integration, and performing its function independently of cell division. They have a transient and controlled expression of the encoded antigen due to a relatively short half-life, and the absence of additional foreign genes ensures their safety. Besides, cell-free manufacture reduces the chances of contamination with bacterial components and facilitates their production under good manufacturing practices. However, RNA vaccines require additional steps in their production and are susceptible to degradation ex vivo and in vivo, while DNA vaccines are more thermostable, facilitating their storage [9,10,11].

One of the main advantages of nucleic acid vaccines over predecessor vaccine platforms is the improvement of the immune response targeting. Besides, this approach allows the addition of antigens from two or more variants in the same vaccine, rapid production, and subsequent modifications to include new variants. Despite initial concerns regarding the possibility of integrating the vaccine plasmid integration into the host’s genome, DNA vaccines have shown remarkable safety, and no significant evidence of integration has been demonstrated [12]. DNA vaccines are composed of synthetic DNA sequences coding an antigen of the targeted pathogen, cloned in expression vectors. After in vivo transfection, the vaccine plasmid needs to reach the nucleus, where the transcription in mRNA will take place, followed by the translation of vaccine antigen peptides in the cytoplasm.

**Figure 1:**
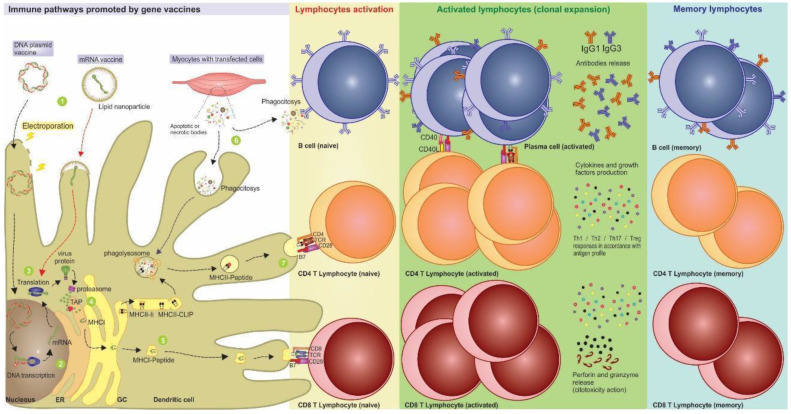
Activation of immunological pathways generated by nucleic acid vaccines. After the vaccine administration, the nucleic acids can be introduced into the dendritic cell through delivery mechanisms such as electroporation and lipidic nanoparticles. (1) Represented by the electric ray, the DNA electroporation facilitates the vaccine entry into the cell through transmembrane destabilization and favors the access of the genetic material to the nucleus and its subsequent transcription. (2) After that, the mRNA is formed and undergoes post-transcriptional modifications, allowing it to escape the nucleus and reach the cytoplasm. (3) The routes for DNA and mRNA vaccines are the same, with the translation of antigen occurring after the endocytosis of the mRNA vaccine. (4) The antigenic proteins processed by proteasomes generate epitopes that are associated with antigen processing (TAP), transported to endoplasmic reticulum, and carried in MHC-I molecules through the Golgi vesicles to be displayed on the cell surface. (5) Thus, MHC-I presenting antigen epitopes and costimulation signals activate naive CD8+ T lymphocytes leading to the production of effector cytotoxic cells, and the induction of immunological memory. (6) Furthermore, exogenous proteins released by transfected cells such as keratinocytes and myocytes can be recognized directly by B cells or phagocytosed by DCs, processed, and presented by MHC-II. (7) In this case, they can activate antigen-specific CD4+ T lymphocytes that expand into differentiated subtypes, release cytokines, and interact with B lymphocytes, leading to a strong humoral response. After antigen stimulus, some lymphocytes migrate to the different lymph nodes as memory cells (or sentinel cells) and are ready for an eventual infection.

After translation, these intracellular antigens are processed inside the proteasome, generating the vaccine epitopes. Then, peptides are transported to the endoplasmic reticulum by the TAP transporter and are linked to MHC-I molecules to be presented to the T cell surface receptors and activate cytotoxic responses. Another pathway of immune response activation can occur from the production and secretion of vaccine antigens from transfected cells, like myocytes. These secreted products are phagocytized by antigen-presenting cells (APCs) and can activate helper responses when presented via MHC-II. The helper response is important because it allows the cross-activation of other cells in the immune system, such as B and TCD8 lymphocytes [13]. More details concerning the activation of immunological pathways by gene-based vaccines are in Figure 1.

## 3. Nucleic Acid Vaccines Allow Better Immune Response Directing

This versatility in activating different immunological response pathways makes nucleic acid vaccines useful for both prophylactic and therapeutic purposes. In this sense, since the main objective of prophylactic vaccines is to promote immunological memory, these strategies demand a strong humoral response through specific CD4+ T cells. A vaccine for therapeutic purposes, on the other hand, requires primarily cytotoxic CD8+ T cell responses to recognize and cause apoptosis of chronically infected or tumoral cells [14,15].

As an example of a prophylactic study, the effects of the mRNA-1273 vaccine against SARS-CoV-2 demonstrated high neutralization and Th1-shifted CD4+ T cell responses in humans. This response profile involved a reduction in the risk of increased vaccine-associated respiratory disease or increasing antibody-dependent replication. Furthermore, these characteristics of mRNA composition and formulation have been associated with prolonged protein expression, induction of antigen-specific follicular T helper cells, and activation of germinal center B cells [16,17].

Regarding the therapeutic approach, Rittig et al. [18] carried out a phase I/II mRNA-based vaccine trial in patients with stage IV renal cell cancer. Direct injection of naked mRNA induced a safe and efficient immune response with specific antitumor immunity promoted by CD4+ T and CD8+ T effector cells. In the study by Cafri et al. [19], an mRNA vaccine for patients with metastatic GI cancer was proven as safe and induced mutation-specific T-cell responses against predicted neoepitopes not detected before vaccination.

Regarding DNA vaccines, two clinical studies tested the VGX-3100 vaccine (NCT01304524) [20] and the GX-188E vaccine (NCT01634503) [21], both encoding immunogenic peptides based on E6 and E7 genes of HPV-16 and 18, in patients with CINs II and III. In the first study, the histopathological results showed regression of lesions in 49.5% of patients (*n* = 53), whereas in placebos, spontaneous regression of lesions occurred in 30.6% (*n* = 11). Furthermore, in this study, the immunological analysis demonstrated significantly greater specific activation of cytotoxic T lymphocytes and increased humoral response in the vaccinated patients [22]. Meanwhile, in the study by Kim et al. [21], 8 out of 9 patients exhibited a polyfunctional response of specific cytotoxic T cells, and 7 out of 9 patients showed complete regression of the lesion, with no viral detection after 36 weeks of follow-up. Due to the COVID-19 pandemic, different platforms have stood out in the face of the public health emergency. DNA vaccines such as the ZyCOV-D vaccine [23] were first licensed in India for emergency use in humans. Others, such as AG0302-COVID19 (NCT04655625), GX-19N (NCT05067946), and INO-4800 (NCT04642638), are currently in phase II/III trials and consist of plasmid vaccines encoding SARS-CoV-2 proteins.

The mechanism by which mRNA vaccines act is like that of DNA vaccines. The main difference is that after immunization, the mRNA vaccines are transported to the cell cytoplasm ready for translation, without the need to reach the nucleus. Besides, the mRNA molecule is less stable and needs to undergo structural changes, such as the addiction of modified nucleosides. Among these modifications is the addition of a synthetic cap on the 5′ region and the poly(A) tail on the 3′ region, needing at least 120 bases to form a mature mRNA sequence. Together, these increments are responsible for increase of translation efficiency, avoiding molecule degradation by cytoplasmic nucleases [11,22,24]. There are two types of mRNA vaccines: non-replicating mRNA vaccines, that encode only the target antigen, and self-replicating RNA vaccines that, besides the antigen of interest, have the replication machinery of positive-stranded RNA viruses such as alphavirus, flavivirus, measles virus, and rhabdovirus, enabling intracellular replication of the vaccine [25].

During the design of a self-replicating mRNA vaccine, the coding sequences of the viral RNA replicase are conserved, while the coding regions of viral structural proteins are replaced by the antigen sequence, preventing the formation of virions in the host. In addition, eukaryotic promoters, such as the CMV promoter, are inserted into the vaccine sequence for recognition by the host’s translation machinery. The action of self-replicating vaccines is like that of conventional mRNA vaccines, except for the fact that after the vaccine transfection, the alphavirus replicase is translated and allows the subsequent replication of more vaccine mRNA molecules. Thus, much lower vaccine doses of self-replicating mRNA vaccines are required to achieve immunizing potential compared to with conventional mRNA vaccines [24,25].

DNA vaccines have been broadly tested in human clinical trials where the immunogenicity, the lack of significant reactions, and the tolerance for doses between 20 µg and 2500 µg have been demonstrated [24,26]. This platform also has high stability at room temperature without the demand for an uninterrupted cold chain for transport and storage, facilitating worldwide access, especially in poor rural areas and tropical countries. Meanwhile, mRNA vaccines have become the focus of different studies, particularly in cancer immunotherapy research, mainly those which use ex vivo modification of antigen-presenting cells [11,27]. Nowadays, this platform has received significant visibility due to the promising results obtained in assays against ebola and H1N1 influenza pathogens [28] and in the face of its extensive use during the COVID-19 pandemic. Furthermore, the first licensed emergency vaccine strategy in the SARS-CoV-2 pandemic was the mRNA vaccine, which retained the highest level of efficacy even after the approval of other vaccine platforms.

Optimizations in the formulation of mRNA vaccines have been sought to maximize their thermostability. An example is the protamine-encapsulated conventional mRNA-based rabies vaccine developed by Sitiz et al. [29]. This study showed the maintenance of vaccine immunogenicity and protective effects through temperature oscillation between 4 and 56 °C per 20 cycles and after prolonged storage (from −80 °C to 70 °C) for several months.

Inside the cell, nucleic acid vaccines can simulate a natural viral infection because they act as an intracellular antigen that can generate specific cellular responses after endogenous production and induce antibody production. Furthermore, the cell transfected with the DNA or mRNA vaccines does not need to be a professional APC to produce the protein antigen capable of stimulating a B or T cell. For example, once expressed by neighboring myocytes, the vaccine antigens can be phagocytosed by APCs and undergo immune cross-presentation [30]. 

The importance of B cells in the efficacy of prophylactic vaccines should not underestimate the role of T-cell responses that are essential for the induction of high-affinity/avidity neutralizing antibodies and memory cells. This role can be explained because the follicular helper cells (Tfh) provide support for the B cell maturation within the germinal centers of secondary lymphoid organs, which can produce high titers of high-affinity and neutralizing antibodies [31]. In addition, activation of helper Th1 response stimulates the secretion of interleukin (IL-2), interferon (IFN-γ), and tumor necrosis factor (TNF-β), with direct antiviral functions and support for cytotoxic T cells and macrophages [32]. In contrast, the Th2 response is suggested as a key factor for the development of vaccine-associated disease enhancement through the production of low-affinity antibodies [33].

The complete activation of both arms of the immune system (humoral and cellular responses) is vital to avoid the lack of affinity maturation of the antibodies. This factor is especially important in the context of COVID-19, since studies have demonstrated the occurrence of vaccine-associated disease enhancement following viral challenge with SARS-CoV, a virus related to SARS-CoV-2 [34]. This issue can be avoided through the careful choice of vaccine antigens, predicted in silico, which must be highly immunogenic and contain MHC-I and MHC-II ligands to activate the cellular response.

Nucleic acid vaccines, especially those with synthetic antigens, allow for the direction of immune response reached by including epitopes recognized by B lymphocytes, MHC-I ligands (cytotoxic response), and MHC-II (helper response) or preferably, all of them simultaneously, in the synthetic construction. One advantage of including T cell epitopes in the vaccine construction is that they can be from any region in the viral antigen, either localized internally or on the protein surface. The recognition of B cell antigens, however, is limited to conformational determinants composed of amino acids located on the surface of the viral antigen. 

## 4. Strategies to Improve the Efficiency of Nucleic Acid Vaccines

Despite their promise, there are still few genetic vaccines approved for use in humans. The main limitations of the production of these vaccines consist of the low immunogenicity inherent in nucleic acid molecules, the challenges related to the transfection of these molecules in vivo, and the instability of RNA molecules. Therefore, there is an effort to enhance vaccine formulation through adjuvants and carriers to increase efficiency [11,35].

### 4.1. Enhancement of Presentation Efficacy of Epitopes to the MHC System

Immunoinformatics is an area that has been increasingly explored in the production of vaccine constructions because it provides several free tools, servers, and databases with information regarding the prediction and analysis of multiple epitopes. Synthetic antigens developed with this approach can be further validated in vivo to provide editable vaccine alternatives that can be updated against emerging variants [36].

From the information contained in immune databases, it is possible, through tools and online servers, to predict and analyze epitopes to be included in prophylactic and/or therapeutic multi-epitope vaccine constructs against infectious agents such as HPV. A study by Sanami et al. included epitopes of HPV16 E6 and E7 oncoproteins in the construction that was suggested as an effective therapeutic vaccine after analysis of antigenicity, allergenicity, and physicochemical properties [37]. Another vaccine for the treatment of cervical cancer was designed with epitopes of HPV16/18 E5 and E7 proteins and showed stability, non-toxicity, and non-allergenicity [38]. Kumar et al. [39] went further, designing a multi-epitope platform with prophylactic and therapeutic potential against HPV16 and 18 from peptides obtained from L1, E5, E6, and E7 proteins that can induce an immune response against CD8+ and TCD4+ lymphocytes. 

During the development of multi-epitope vaccines, after a careful selection of immunogenic epitopes and their arrangement in the vaccine construction, it is necessary to ensure that the immunogens are passive to be translated and presented through the corresponding MHC on the APCs’ surface. A strategy to increase vaccine gene expression is the optimization of species-specific codons. Studies have shown that codon optimization can lead to increased cellular and humoral immune responses [40,41]. Moreover, a concern during the development of a multi-epitope vaccine is the possibility of losing ‘natural flanking sequences’ that could impair the correct individual cleavage of epitopes through the proteasomal and lysosomal pathways.

Studies have shown that the flanking residues of MHC-I binding epitopes have a powerful influence on its appropriate processing by the proteasome and, consequently, on its presentation. Livingston et al. [42] analyzed 94 different epitope/flanking region combinations and discovered that the type of residue located immediately following the carboxyl terminus could affect its immunogenicity. This study found that high levels of immunogenicity were correlated with the presence of basic, amide, or small residues at this extremity. In contrast, low levels of immunogenicity were associated with the presence of aliphatic or aromatic residues. Thus, it became possible to modulate the immunogenicity of each epitope in the vaccine construction by including efficient flanking regions among them [43].

**Figure 2:**
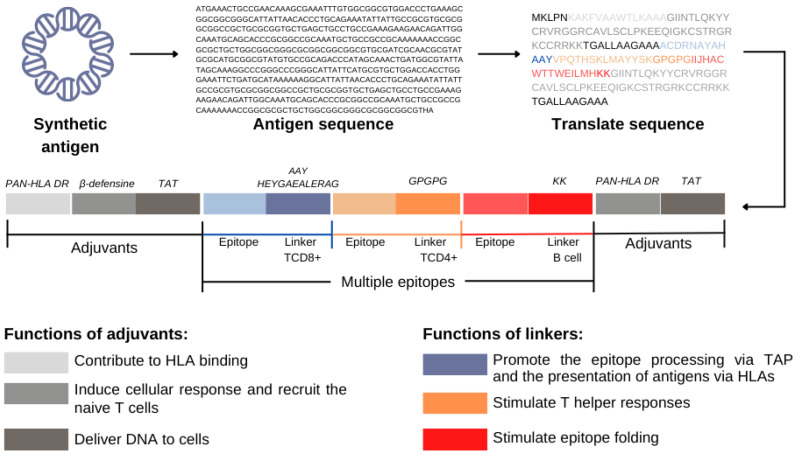
Functions of adjuvants and linkers in the synthetic antigen. They are sequences that help to cleave the peptide formed and stimulate the immune response, respectively.

Another approach is the addition of spacer sequences between the epitopes [44]. Such sequences are known as linkers and have been specially engineered to offer proteasomal and lysosomal cleavage sites, as well as binding sites to the TAP transporter, thus increasing the efficiency of MHC pathway presentation. Some examples are the motifs HEYGAEALERAG, AAY, GPGPG, and KK (Figure 2) [30,34,45,46].

### 4.2. Increasing Vaccine Immunogenicity

Despite encouraging results in previous preclinical tests, DNA vaccines have shown limited immunogenicity in superior primates and humans [47]. This limitation can be due to the inefficient uptake of naked plasmid DNA. In general, the plasmid can become trapped in the extracellular space after the administration and is susceptible to rapid degradation by endonucleases. Thus, the low amount of antigen available at the site of administration results in low uptake of target DNA by cells, which impacts the number of molecules to be transcribed and subsequently translated into vaccine antigens [48]. Therefore, adjuvant administration is essential to overcome the suboptimal efficacy presented by most genetic vaccines [49]. Adjuvants can act in many ways, such as increasing antigen presentation (depot formulation, delivery systems); some examples are β-defensine, PAN-HLA DR, and TAT [34]. 

An important class of adjuvants is the toll-like receptor (TLR) ligands. TLRs are a family of receptors found on the surface (TLR1, TLR2, TLR4, TLR5, and TLR6) and in endosomes (TLR3, TLR7, TLR8, and TLR9) of immune cells. These receptors can quickly identify conserved molecular features of pathogens, the pathogen-associated molecular patterns (PAMPs), identifying them as “dangerous” and enhancing the production of the pro-inflammatory cytokines. Moreover, activating the innate immune response, these receptors increase the presentation of antigens to lymphocytes by dendritic cells (DCs) [50]. 

DCs cells play a central role in activating cell response through the antigen presentation to TCD4 and TCD8 lymphocytes in the draining lymph nodes. In the lack of danger signals, DCs cannot properly stimulate T lymphocytes due to the absence of costimulatory molecules (CD80/CD86 and CD40) on their surface [50]. In addition, TLRs contribute to the direct activation of B lymphocytes during T cell-independent antibody response (through B cell receptor [BCR]) and indirectly during T cell-dependent response (acting with Tfh cells within the germinal centers). Thus, these receptors integrate innate and adaptive immunity and are an excellent target for vaccine adjuvants [51,52].

The endosomal TLRs detect nucleic acids of viral and bacterial origin and can generate cytotoxic responses to eliminate viral pathogens and cancer cells [53]. In addition, the TLR3, TLR4, TLR7, TLR8, and TLR9 signaling pathways promote Th1 responses [54], whose lymphocytes release TNFα, IFNγ, and IL-12, stimulating B cells to produce high levels of IgG and IgA antibodies for pathogen elimination. TLR9 is particularly interesting for use in the context of DNA vaccines, whose detection of non-self DNA occurs by the presence of unmethylated CpG motifs [55]. An important class of TLR9 agonists widely used as a vaccine adjuvant is the synthetic oligonucleotides (ODN) composed of CpG motifs, capable of inducing strong cytotoxic responses [56]. 

Another promising example of an adjuvant molecule is the TLR-3 agonist, the polyriboinosinic polyribocytidylic acid [Poly(I:C)]. This molecule consists of a double-stranded RNA analog capable of inducing cell signaling through multiple inflammatory pathways. Poly(I:C) is especially used in formulations that target dendritic cells, promoting their maturation [55]. In a study using a DNA vaccine, the combination of both CpG/poly (I:C) adjuvants exhibited significantly stronger IFN-γ responses and generated high levels of CD4(+) response for cytokines IL-2, IL-4, and IFN-γ, and a CD8(+) response for cytokines IL-2 and IFN-γ [57]. Another study found that poly (I:C) adjuvants and resiquimod, a toll-like receptor 7 (TLR7) agonist, induced significant tumoral regression in a therapeutic DNA vaccine encoding the E7 gene of HPV-16. This study demonstrated the induction of IFN-γ and nonspecific intratumoral IL-12 in mice towards a Th1 immune profile [58]. Similar results were obtained by Öhlschläger et al. [59] using a CpG cassette into the plasmid backbone of a therapeutic DNA vaccine followed by electroporation (EP).

In addition to TLR ligands, many other classes of immunomodulators can be used through co-injection or even as part of the vaccine construction in nucleic acid vaccines. Cytokines are a class of immunoregulatory proteins critical to the signaling of immune cells and capable of affecting their behavior. Several studies have included plasmids encoding ligands of growth factors, adhesion molecules, death receptors, and other cytokines [60]. An example is the use of interleukin-2 (IL-2) due to its essential role in the differentiation of naïve T cells into effective T cells and memory cells [59]. Studies with DNA vaccines that included IL-2 demonstrate a significant increase in immunogenicity for influenza [36], SARS-CoV [49], and HIV vaccines. Among these studies, the one using an HIV-1 DNA vaccine which included IL-2 and the Fc portion of immunoglobulin G (IgG) described a potent increase in cellular response in rhesus monkeys [61].

IL-12 is another cytokine that provides a link between innate and adaptive immunity in response to infections [62]. Furthermore, IL-12 supports the expansion of activated Th1 cells, increasing the cytotoxic response and its mediators, such as IFN-γ, granzyme B, and perforin, which are key factors for intracellular pathogen clearance [63,64]. Many studies involving the inclusion of IL-12 in expression plasmids have demonstrated a highly immunogenic effect of this adjuvant, with high IFN-γ production and high levels of cytotoxic and helper response. Most studies combined the IL-12 actions with electroporation in DNA vaccines [51,52,62].

In addition to establishing the profile of these adjuvants, it is necessary to assess how they will be delivered and presented to immune system cells. Many of them are inserted in DNA plasmids, encoding sequences of inflammatory cytokines, chemokines, interferons, and growth factors, and are co-administered with the nucleic acid vaccine [65]. Others are cloned on the same plasmid as the gene of interest through the incorporation of IRES or T2A sequences that allow the co-expression of antigens or adjuvants in the same vector [66]. Despite substantial recent improvements, the most appropriate way of distributing the available adjuvants remains controversial.

Lapuente et al. [67] observed that the co-administration of H1N1 antigens and plasmids containing a constitutively active version of RIG-I, IPS-I, IL-1, or IL-18, followed by electroporation in mice, did not affect the efficiency of a DNA-based influenza A vaccine. The co-administration of pDNA encoding GM-CSF, Flt-3L, and IL-12 alone or in combination, on the other hand, boosted the activity of HIV plasmid DNA vaccines [68]. Otherwise, Kumari et al. [69] created a bicistronic vaccine including an IRES sequence between the IFN-γ and glyceraldehyde-3-phosphate dehydrogenase genes that maximized the protection against *Edwardsiella tarda* infection induced.

### 4.3. Vaccine Transfection Efficacy Enhancement

The type of vaccine platform represents an important aspect regarding the intensity of the associated immune response. Vaccines that contain live virus, for example, accurately simulate the natural process of infection and, after administration, quickly disseminate through the circulation, reaching their target tissues. These viral particles promote a broad response and contribute to the transmission of signals associated with pathogens, which mobilizes the innate immune system response. Meanwhile, non-live vaccines, such as nucleic acid vaccines, exhibit limited immune response due to the lack of replicative activity, essentially activating the innate immune response locally at the point of administration. In this case, the site and route of administration are relevant factors of choice [70].

For nucleic acid vaccines, the most common routes are intradermal and intramuscular due to the availability of DCs in these tissues that allows a successful immunization without the need for high antigen doses, especially under limited immunogenicity conditions. Another alternative route for non-live vaccine injections is the vascularized muscle tissue because it has a region with high amounts of DCs. Some routes may be less effective, such as subcutaneous injections, since there is a lower distribution of these cells in adipose tissue [71].

Whereas the intramuscular route (IM), followed by electroporation, is pointed as the best administration route for DNA vaccines [72], for mRNA vaccines, intradermal (ID) injection seems to be more appropriate [73]. This difference may be due to the cationic nature of the liposomes commonly used to carry the mRNA vaccine, which depends on the size and charge properties for its mobility in the muscle fibers and consequently interferes with the nanoparticle distribution in DCs. Therefore, positively charged and smaller particles (<50 nm) could be applied to improve mobility when using this administration route [74]. However, some results are controversial and may depend on other factors, such as the vaccine doses or adjuvants, since a study has shown that a DNA vaccine was more immunogenic through the ID route than the IM route [75]. Thus, more studies are necessary to obtain conclusive results. 

In addition to the immunization route, it is essential to ensure that nucleic acid vaccines are transported through the biological barriers and can be delivered inside the cell before they become degraded. Hence, improving delivery systems for nucleic acid vaccines constitutes an important research area for vaccine development. In this review, we focused on the electroporation (EP) for DNA and lipidic nanoparticles (LNPs) for mRNA vaccines, which are the most common systems employed to increase the immunogenicity of acid nucleic-based vaccines.

Over the years, electroporation has been applied in several preclinical and clinical trials to improve the delivery of nucleic acids and chemotherapeutic drugs to target tissues. This technique emerged as an introductory method for macromolecules and was associated with an increased expression of these molecules in vivo [47]. The proposed action mechanism consists of transmembrane destabilization through the application of electric pulses, which results in transitory pores that will allow the direct passage of DNA into the cell cytoplasm [76]. During the process, an electrophoretic effect is responsible for transporting the DNA to the nucleus, increasing the plasmid delivery and cell transfection efficiency. Cappelletti et al. [77] showed that a large part of the inserted DNA is degraded approximately four hours after administration. In this sense, EP contributes to the increase in the efficiency of immunization since it allows a higher plasmid uptake from tissue cells. Furthermore, studies have demonstrated the presence of necrotic and apoptotic bodies in the inflammatory environment generated after electroporation. The cell debris containing vaccine peptides can be encompassed by DCs, with the activation of helper response [78].

A study demonstrated that electroporation modulated the production of pro-inflammatory cytokines in the skin, elevating local concentrations of transforming growth factor-α (TGF-α) and IL-1 [79]. Moreover, EP allows the recruitment and interaction of Langerhans cells with the transfected cells before being directed to the epidermis [80]. Preliminary reports in clinical trials have shown successful delivery of tumor antigens after EP in immunotherapy approaches [81]. Vasan et al. [79] showed that EP corresponds to a safe technology capable of eliciting the increase, durability, and amplitude of the immunogenicity of the DNA vaccine.

Diken et al. [82] showed that the RNA encapsulated in LNPs was efficiently internalized by murine and human DCs conducted by macropinocytosis, from in vitro and in vivo studies. However, in addition to proper cellular delivery, it is also important to ensure the escape of the mRNA from endosomes. Hence, some strategies have been developed, such as those using liposomes based on cationic lipids, which are pH-sensitive lipids that become fusogenic in a slightly acidic medium (6.5–5.0). An example is the MM27, a lipid composed of an imidazole group that is protonated at acid pH. This process increases the liposome’s fusogenic properties and promotes its destabilization and cytosolic distribution. Many other lipids with different compositions have been studied to improve the in vivo transfection of genes such as 1,2-dioleoyl-3-trimethylammonium-propane (DOTAP), 1,2-distearoyl-sn-glycero-3-phosphocholine (DSPC), 1,2-dimyristoyl-sn-glycerol, methoxypolyethylene glycol-2000 (DMG-PEG2000) [74]. 

Miao et al. [83] used a combined data system, including various lipid formulations, to identify possible mechanisms that facilitate mRNA delivery, providing a robust and specific immune response. The major lipidic candidates showed some structures in common, such as an unsaturated lipid tail, a dihydroimidazole ligand, and cyclic amine groups. These formulations were able to induce APC maturation and resulted in limited systemic cytokine expression, as well as increased antitumor efficacy.

## 5. Conclusions

Third-generation vaccines represent a huge advance in the field of vaccinology. The COVID-19 pandemic reinforced the importance of this vaccine platform, providing an excellent example of how the pioneer studies initiated decades ago could be applied in an extremely fast, efficient, safe, and cost-effective way—as never seen before—in the fight against the pandemic. This review has compiled research trends on the engineering of third-generation vaccines and the various aspects that can be addressed to increase their efficiency. As presented here, growing knowledge about the immune system has supported the bioengineering of molecules capable of activating immune responses with higher efficiency, specificity, and safety. The challenge now lies in further improving this technology to overcome its limitations, such as the low immunogenicity of DNA molecules and the inherent instability of the RNA molecule. In this regard, recent studies are developing new generations of biomolecules for vaccine delivery, as well as new adjuvants.

In addition to the advances described in this work, it is worth mentioning the increasing number of studies that feed the international databases that, in turn, support bioinformatics tools, allowing future assistance in the development of new tools, in addition to the improvement of existing ones. All of this will contribute to increasingly accurate epitope predictions during the vaccine construction stage. Furthermore, thanks to next-generation sequencing, a massive deposit of genomic sequences has been observed, which in turn will contribute to the characterization of MHC profiles of different populations in the world, allowing the development of multi-epitope vaccines with greater population coverage capacity. All these advances in studies and techniques discussed here lead to a positive expectation for the future of vaccinology, helping to ensure the resolution of infections associated with challenging pathogens or even complex and multifactorial diseases such as cancer.

## Data Availability

Not applicable.
